# The Predominant Oral Microbiota Is Acquired Early in an Organized Pattern

**DOI:** 10.1038/s41598-019-46923-0

**Published:** 2019-07-22

**Authors:** Rosalyn M. Sulyanto, Zachary A. Thompson, Clifford J. Beall, Eugene J. Leys, Ann L. Griffen

**Affiliations:** 1 Boston Children’s Hospital and Harvard School of Dental Medicine, 300 Longwood Avenue, Hunnewell 4, Boston, MA 02115 USA; 2 The Ohio State University, College of Dentistry, 4126 Postle Hall, 305 W. 12th Ave, Columbus, OH 43210 USA

**Keywords:** Microbiome, Microbial ecology, Microbiome, Microbial ecology, Microbiome

## Abstract

The human oral cavity is sterile prior to birth, and we have limited knowledge of how complex oral communities are assembled. To examine bacterial acquisition and community assembly over the first year of life, oral samples from a cohort of nine infants and their mothers were collected, and bacterial community composition was studied by 16S rRNA gene sequencing. Exogenous species including skin and environmental bacteria were present initially, but were quickly replaced by a small, shared microbial community of species common to all infants and adults. Subsequent ordered microbial succession and the formation of increasingly complex communities was observed. By one year of age oral microbial community composition converged to a profile that was remarkably similar among children. The introduction of new nutrient sources, but not tooth eruption, was associated with increasing complexity. Infants had fewer species than mothers, mostly accounted for by the lack of certain anaerobes, and showing that the acquisition and assembly of oral microbial communities continues past infancy. When relative abundance was considered, a shared set of species accounted for the majority of the microbial community at all ages, indicating that the dominant structure of the oral microbiome establishes early, and suggesting that it persists throughout life.

## Introduction

The individual adult human oral cavity typically hosts more than 200 species of bacteria, and over 600 species are commonly found in the broader adult population^1,2^. The oral cavity is sterile prior to birth^3^ and we have limited knowledge of how complex human oral communities are assembled, or when key species are acquired. The timing and sequence of acquisition has been studied, but only by cultivation, or for a few species using targeted methods. In the first few minutes after birth the neonatal oral cavity has been shown to harbor microbiota resembling vaginal or skin microbiota, depending on mode of delivery^4^. A 16S rRNA gene-based study with resolution at the genus level showed that by the age of 4 months complex communities containing organisms typically present in adults are detected, including *Streptococcus*, *Veillonella*, and *Neisseria* species^5^. Other studies on the infant oral microbiome have shown a lower diversity in the infant oral microbiome compared to older ages as well as effects of maternal smoking, delivery mode, and breast feeding^6,7^. Older cultivation-based studies have tracked the acquisition of key species, including *Streptococcus mitis*, an early colonizer^8,9^, and *Streptococcus sanguinis*^10^, a late colonizer. Multiple studies have explored colonization with the caries pathogen *Streptococcus mutans*, with some studies demonstrating its presence only after tooth eruption^11^ and others detecting it in the mouths of pre-dentate infants^12^. More comprehensive cultivation-based studies have tracked the sequential assembly of early oral bacterial communities^13^, and have suggested microbial dependencies and succession. However, these studies did not provide the resolution and comprehensiveness of newer 16S-based approaches, or provide a clear outline of the development of the infant oral microbiota.

The acquisition and assembly of human oral microbial communities has potentially important implications for health and pathogenesis, since differences in microbial community composition are well correlated with human microbial diseases such as periodontitis and dental caries^14–18^. A better understanding of how oral microbial communities are assembled may ultimately aid in the development of preventive and therapeutic strategies to guide the formation of health-promoting bacterial communities.

To examine oral bacteria acquisition and community assembly over the first year of life, we collected samples from a cohort of nine healthy infants and their mothers and studied their oral communities by 16S rRNA gene sequencing.

## Results

Nine mother-infant dyads participated in this study. All infants were delivered at full term with no medical complications and had their biological mother as their primary caregiver. Saliva samples were collected up to monthly from 0 to 12 months of age for infants and at baseline for mothers. Six of the child participants were female. Five of the children were vaginal deliveries and 4 Caesarean. Self-reported race was 3 African American, 3 Caucasian, and 2 Asian. Three children were solely breast fed, one solely formula fed, and five breast and formula fed.

Out of 117 possible child samples 69 were collected. 25 samples were collected from the mothers. A total of 575,597 sequences passed quality and length filtering. After filtering for chimeras, 516,993 sequences were assigned to an OTU. Samples with low sequence totals ( < 100) were not included in further analyses, removing three child and one mother sample. Ninety samples then remained for analysis, averaging 5,744 sequences per sample and with quartiles: min = 103, Q1 = 1068.5, M = 2313, Q3 = 4850, max = 86351.

**Figure 1:**
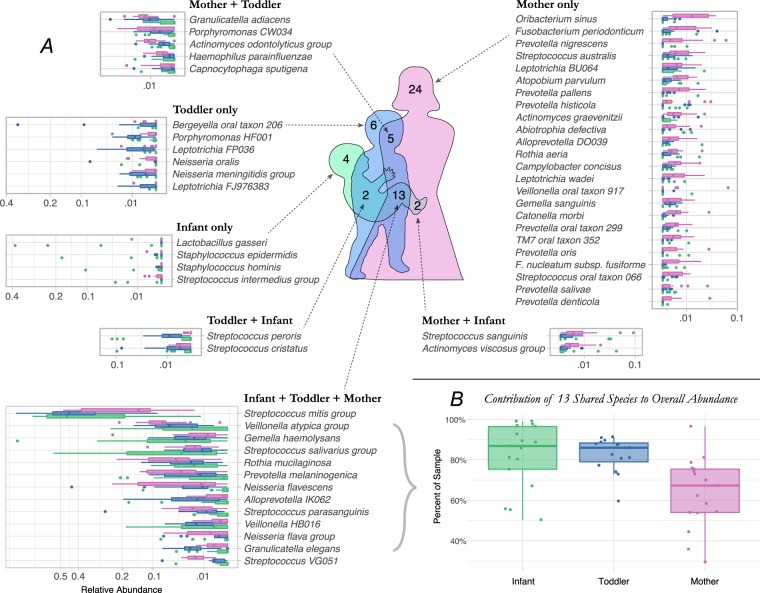
Oral microbial communities in mothers, infants, and toddlers. (**A**) Venn diagram and relative abundance boxplots for species detected in mothers, infants and toddlers are shown. Number of species detected for each Venn intersection are shown within the diagram, and species are listed for each intersection. For each species, boxplots show square root-adjusted relative abundances for infants (green), toddlers (blue) and mothers (pink). Only species that met empirical probability thresholds were included. (**B**) The contribution to total microbial relative abundance of those 13 species present at all ages are shown in boxplots.

**Figure 2:**
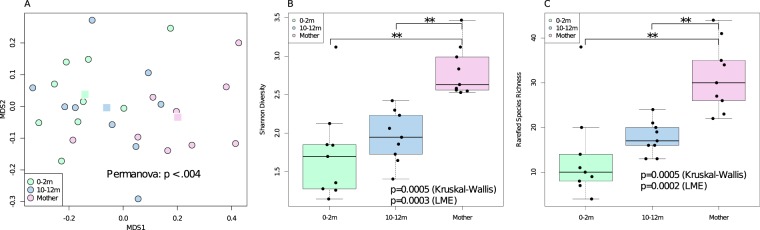
Comparison of microbial community composition in infants, toddlers and mothers. (**A**) Overall composition differed among infants, toddlers and mothers, as shown in a Bray Curtis Multidimensional Scaling plot where centroids are shown as square points. Multiple samples from a single individual were combined within each age range. A PERMANOVA analysis showed overall differences between the groups while *post hoc* PERMANOVAs showed significant differences between mothers and either of the baby groups (mothers:0–2 month p = 0.0039; mothers:10–12 month p = 0.078). (**B**) Shannon diversity and (**C**) richness varied significantly among age groups (by Kruskal-Wallis), and increased with increasing age (by LME). Bars indicate significant *post hoc* comparisons (Mann Whitney test) with the two asterisks showing p values between 0.01 and 0.001. The mean number of species detected per infant from 0–2 mo of age was 30, from 10–12 months it was 33, and in mothers it was 83 (95% CI = 14–47, 15–50, 64–102, respectively).

A Venn diagram of the species detected in the saliva of mothers, and children as infants (age 0–2 months) and as toddlers (age 10–12 months) is shown in Fig. 1A. A total of 331 species were detected at least once, but many of these were uncommon. Only species that met empirical probability thresholds (described in methods) were included in the figure. Community membership for all intersections and non-unions are shown in Fig. 1A, and relative abundance plots by species are shown. The overall microbial community composition as determined by Bray Curtis Multidimensional Scaling varied significantly between children and mothers (Fig. 2A), and the complexity and richness increased with increasing age (Fig. 2B,C). Despite these differences in community membership, when relative abundance was considered, over two-thirds of the microbial community in mothers was composed of the set of 13 species shared with infants and toddlers (Fig. 1B).

**Figure 3:**
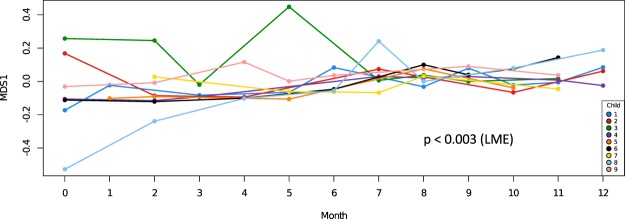
Similarity of microbial community composition among children over the 1^st^ year of life converged. Using the first dimension of a non-metric Multidimensional Scaling (MDS) based on total microbial community composition, the distance to the centroid was plotted against time and analyzed using a linear mixed effects model (p < 0.003).

Figure 3 shows that microbial community composition among children was most variable in the early months of life and converged to a more similar, shared profile with increasing age (p = 0.003).

**Figure 4:**
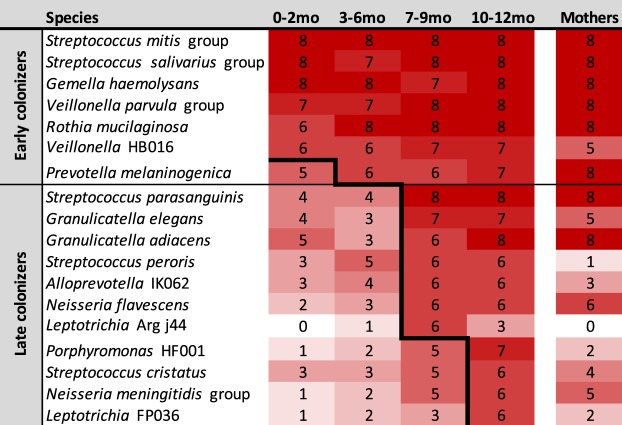
Species were acquired in an ordered sequence. A heatmap based on prevalence of all species found in ≥50% of subjects is shown. Monthly samples were collapsed into 3-month intervals for each child, and rarefied as described in methods. Species are sorted by decreasing overall prevalence, and the stepped heavy line demarcates the age at which they were detected in ≥75% of children.

The pattern of community assembly with sequential species acquisition is shown in Fig. 4, a heatmap of the prevalence of the most common species at intervals between birth and age 1 year. Multiple samples for each child were combined within the 3-month intervals. One child was excluded because too few sequences were available for the 3–6 month interval. The *Streptococcus mitis* group (*S*. *mitis*, *S*. *infantis*, *S*. *pneumoniae* and *S*. *oralis* are not distinguishable by 16S rRNA gene sequence) was detected in the earliest samples from 100% of infants, and remained ubiquitous at all ages. Within the first 2 months of life 6 species including the *S*. *mitis* group were present in ≥ 75% of infants. An additional species was added by 6 months of age, a third group of 7 newly acquired species reached ≥ 75% prevalence by 9 months of age, and by 12 months of age, an additional 4 species were present in ≥ 75% of infants (Fig. 4). Most common species in infants were also highly prevalent in mothers, with the exception of *S*. *peroris*, uncultivated *Leptotrichia* Arg j44, and *Porphyromonas* HF001.

**Figure 5:**
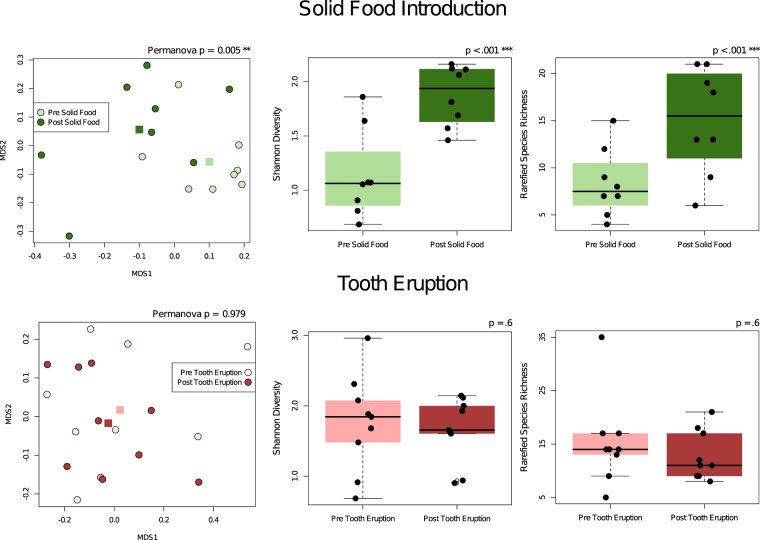
Introduction of solid food but not tooth eruption had a significant effect on microbial community composition. Time of tooth eruption was recorded, and samples taken immediately pre- and post- tooth eruption and pre- and post- introduction of solid foods were included in the analyses. On average infants began solid foods at 6.8 ± 0.9 months of age and tooth eruption at 8.7 ± 2.2 months. Bray Curtis Multidimensional Scaling plots with group centroids plotted as square points and box plots for richness and diversity are shown (analyzed by Wilcoxon Signed Rank Test).

Significant changes in microbial community composition, diversity and richness were observed after introduction of solid foods, but not after eruption of the first tooth (Fig. 5). Species-level analysis showed *S*. *mitis* becoming a significantly lower fraction of the total community, but no single species showed significantly higher abundance.

## Discussion

This study profiled the assembly of the oral microbiota at the level of species from birth through the first year of life using 16S rRNA gene analysis. As expected, a comprehensive, longitudinal analysis showed that the infant and toddler microbiota consisted of a subset of the adult community (Figs 1 and 2). Despite this difference in community size, when relative abundance was considered, the shared set of species present in both children and mothers accounted for over two-thirds of the microbial community at all ages (Fig. 1B), demonstrating that the dominant community structure of the oral microbiota establishes very early, and suggesting that it is stable throughout life.

This study also revealed that the oral microbiota is acquired in an ordered sequence and time course that was shared among infants (Fig. 4). This has previously been observed in the human infant gut, where the phylogenetic diversity increases gradually over time, and community assembly occurs as an orderly succession^19^ that ultimately resembles the profile of the adult gastrointestinal tract^20^.

Within the first 3 months the majority of infants harbored a simple core microbial community of six species/groups: the *S*. *mitis* group, *Rothia mucilaginosa*, the *Veillonella parvula* group, the *Streptococcus salivarius* group, *Gemella haemolysans*, and *Veillonella* HB016 (Fig. 4). The most prevalent species detected in young infants in this study confirmed those reported in a recent survey of the oral microbiota in 3-month old infants^21^. Additional species increased in prevalence between 3 and 6 mo of age, including members of the *Prevotella*, *Granulicatella*, and *Neisseria* genera (Fig. 4), suggesting a dependence on earlier colonizers. Most of these are common in adults, but some species were more common in the infants than in mothers, including *S*. *peroris*, and uncultivated *Leptotrichia* Arg j44, *Porphyromonas* HF001, and possibly *Leptotrichia* FP036. The high prevalence of these species in children has been confirmed in a larger, unpublished study (data not shown), suggesting that developing oral communities may contain members whose role is temporary.

In this study very young infants harbored some species not commonly detected in the human oral cavity (Fig. 1), including staphylococci commonly detected on human skin. Other transient species found at levels too low to meet thresholds for inclusion in Fig. 1 included gut and environmental species. Despite the early presence of transients, microbial community composition among children converged to a similar, shared profile within a few months (Fig. 3), as species poorly adapted to the oral cavity were lost and a shared core set of commensal species replaced them. This selection is remarkable considering the nutrient-rich aqueous environment and open nature of the oral cavity. Exposures to environmental microbes from water and soils attached to foods, microbes from plant, meat and dairy foods, pets, and airborne microbes are unrelenting, yet colonization is tightly limited. The mechanism for this exclusion may be a combination of host innate and adaptive immune functions, along with colonization resistance by commensals. This may provide an important protective function, and the host-microbe interactions that drive this deserve further study. The *S*. *mitis* group was the earliest and most ubiquitous colonizer, and has been shown to inhibit the colonization of other species, including potential pathogens^22^.

The possibility that the non-oral species detected in the earliest samples were the result of low DNA yields and reagent contamination was considered, but we think it unlikely. Self-sampling may also have contributed to variation, since after initial training, mothers sampled their own children. The decontam R package^23^ did show 3 species with p-values of less than 0.05, but none were among the non-oral species noted in the samples from very young infants. For analyses that depended on presence/absence, counts were rarefied. This was done in part to allow inclusion of low count, low microbial biomass samples without overamplifying them, and also to minimize inclusion of species represented at such low levels that they could be transient or contaminants. Although approximately 500 sequences were ultimately tabulated for each sample, they were determined by a probabilistic model derived from a much larger number of sequences (average = 5,744 per sample).

Potential mechanisms for the shared pattern of acquisition of bacterial species over the first year of life (Fig. 4) are only partially understood. Bacteria are unlikely to survive as planktonic inhabitants of saliva, because the constant flow into the gastrointestinal tract will overcome their ability to reproduce, so adhesion to surfaces is an important requirement for colonization. Tooth enamel is not present in early infants, so the organisms must adhere either to epithelial tissues or other microorganisms in a secondary interaction. Adhesion mechanisms in some of the early colonizers have been elucidated, and could be relevant to the process of early colonization. The ability of *S*. *mitis* and *S*. *salivarius*, which we identify as important early colonizers (Fig. 4), to bind to mucosal surfaces in infants has been previously demonstrated^24^. *S*. *mitis* amylase binding proteins have been shown to bind salivary amylase and facilitate adherence of *S*. *mitis* and other streptococcal species^25,26^. Members of the *S*. *mitis* group of species contain serine-rich repeat proteins on their surface^27,28^ that bind to a variety of partners, including fibronectin, cells, and other bacteria^27^. Interbacterial adhesion has also been demonstrated and is likely to contribute to the order of acquisition. Lectin-mediated binding of many oral streptococci to other oral bacteria has been shown to occur via a family of antigenically diverse cell wall polysaccharides^29^. Highly specific coaggregation has been observed among the early colonizers identified in the present study, *Streptococci*, *Veillonella* and *Rothia*^20,30,31^. Mechanisms may include surface structures important for biofilm formation in *R*. *mucilaginosa*^32^, and a *V*. *atypica* (part of *V*. *parvula* group) adhesin, Hag1, that can mediate binding to both oral epithelial cells and other bacteria^33^. In addition, other mechanisms including nutrient dependencies, host immune factors, and bacterial antagonistic strategies are likely to be important. A recent study of bacterial growth on saliva is possibly relevant to the succession that we observe in the infant mouth^33^. In this work, members of the early colonizing *Streptococcus mitis* group, *S*. *mitis* and *S*. *oralis*, were able to grow well in saliva, which correlated to their ability to cleave sugars from salivary proline-rich glycoproteins (PRGs). The early colonizer *G*. *haemolysans* grew at an intermediate level, as did the late colonizer *Granulicatella elegans*, while the late colonizers *Streptococcus parasanguinis* and *Granulicatella adiacens* grew poorly. All of these species also failed to hydrolyze the sugars from PRGs^33^. It is possible that the early colonizers provide a food source to the later colonizers by cleavage of the carbohydrate components of these glycoproteins, and that might explain the order of acquisition we observe.

Introduction of solid foods typically occurs in close proximity to tooth eruption, and effects on community composition have not been separated in previous studies. In our sample, a significant increase in oral microbial diversity was observed after introduction of solid food, but not after the eruption of teeth (Fig. 5). Acquisition of new species around this time has been attributed to the availability of tooth surface for attachment^12^, but new substrate may be more important. Studies of the infant gut microbiota have shown that ingestion of solid foods results in an abrupt shift that ultimately results in a more stable community composition characteristic of the adult gut microbiota^19^. In a comparative analysis of the salivary bacterial microbiota of predentate infants and their mothers, the exposure of an infant to solid foods was associated with the infant’s oral microbial community composition clustering more closely to adults than to other infants^5^. These findings corroborate the contribution of diet to the development of the oral microbial community. Samples were collected from teeth once they erupted, but DNA yields were almost an order of magnitude lower than from the saliva swabs, and too low to reliably amplify bacterial 16S rRNA genes. This suggests that tooth-associated communities are slow to develop, and could explain the lack of immediate bacterial community shift on eruption.

The earliest colonizers are predominantly aerobic or facultative bacteria except for *Veillonella*. V. parvula has been shown to make catalase, and provide protection for more fastidious anaerobes^34^, and may provide the environment that allowed the succession of several anaerobic species that was observed in the second 6 months of life (Fig. 4). This may occur as maturing biofilm communities develop micro-niches protected from oxygen. These species level findings are consistent with previous genus-level reports on anaerobes in the infant oral cavity that showed *Veillonella* as one of the predominant anaerobes in infants^35^, and that *Prevotella*, *Porphyromonas*, *Leptotrichia*, *and Actinomyces* species colonize infants by 1 year of age^36^. Species found exclusively in mothers (Fig. 1A) were lower abundance, predominantly anaerobic species whose niche may be a deep gingival sulcus not yet developed in young children. Some of these anaerobes have been associated with periodontal diseases and systemic infections that are rare in young children. As we continue this study to include children beyond 1 year of age, we aim to examine the relationship between the acquisition of anaerobes and the deepening of the periodontal sulcus that occurs with both age and periodontitis.

No significant differences in microbial community profile were observed for sex, race, mode of delivery, feeding mode or antibiotic use, and infants did not match their own mothers more closely than unrelated mothers (data not shown), but sample sizes and power to detect differences were small. A recent study found changes in bacterial communities of infants due to delivery mode at early times and breastfeeding at later times^6^.

## Conclusions

This longitudinal study of infants and their mothers profiled the development of the oral microbiota from birth to 12 months of age. Exogenous species including skin and environmental bacteria were present initially, but were quickly replaced by a small, shared microbial community of species common to all infants and adults. Subsequent ordered microbial succession and the formation of an increasingly complex community was observed. By one year of age oral microbial community composition had converged to a subset of the adult oral microbial profile that was remarkably similar among children. The introduction of new nutrient sources, but not tooth eruption was associated with increasing complexity. Infants had fewer species than mothers, mostly accounted for by the lack of certain anaerobes. This shows that the acquisition and assembly of oral microbial communities continues past infancy, and that studies should be carried forward to older age groups. When relative abundance was considered, at all ages a shared set of species accounted for the majority of the microbial community, indicating that the dominant community structure of the oral microbiome establishes early and suggesting that it persists throughout life.

## Methods

### Subject recruitment

Mother-infant pairs were recruited from Nationwide Children’s Hospital, Columbus, Ohio, and The Ohio State University for this Nationwide Children’s and Ohio State University IRB-approved study, the subjects or their guardians gave written informed consent, and the study was carried out according to relevant institutional guidelines and regulations. Inclusion criteria for infants were full-term delivery, ASA I or II status, and an English- speaking biologic mother as a primary caregiver.

### Sampling and clinical data collection

Infant and maternal saliva samples were collected up to monthly for twelve months, with a baseline sample taken within 2 weeks after birth. Mode of birth delivery, race, and ethnicity were recorded at baseline. Infant feeding practices, antibiotic use and onset of teething were recorded at each sampling. Saliva samples were collected from infants by placing a small, sterile, flocked swab (Copan Diagnostics Inc., Murrieta, CA) in the lingual vestibule for 30 seconds to saturate it. Saliva samples were collected from mothers by expectoration of unstimulated saliva into a 50 ml tube. The initial sample was collected by mothers under the direct supervision of RS, a dentist, and mothers were instructed to perform collection the same way for subsequent sampling. Sampling packets with written instructions, sterile swabs, and tubes containing DNA stabilizing solution (Buffer ATL, QIAGEN, Germantown, MD) were mailed to mothers at monthly intervals. Samples were collected at home by mothers and mailed back to investigators within one week. Upon arrival in the laboratory samples were immediately frozen until time of DNA extraction.

### 16S rRNA Gene Amplification and sequencing

Bacterial community composition was determined by 16S rRNA gene amplification and sequencing. DNA was isolated from samples using QIAmp DNA Mini Kits (QIAGEN, Valencia, CA) following the manufacturer’s protocol with the addition of a bead-beating step to increase recovery from difficult-to-lyse bacterial species. The V1 to V3 region of the 16S rRNA gene was amplified with the HMP primers containing bar codes, Broad Institute versions^37^. Amplicons were purified using AMPure XP magnetic beads (Beckman-Coulter, Indianapolis, IN), quantitated with the Quant-iT kit (Invitrogen, Eugene, OR), pooled in equimolar quantities, purified a second time by AMPure XP, and finally quantitated by real-time PCR (Kapa Laboratories, Woburn, MA). Pools were sequenced on the Roche 454 GS FLX instrument (Roche, Branford CT) using the HMP V1V3 454 sequencing protocol (https://www.hmpdacc.org/hmp/doc/16S_Sequencing_SOP_4.2.2.pdf). The approximately 500 bp amplicon was sequenced in a single read.

### Bioinformatics methods

Sequences were separated by barcodes, trimmed for primers and low quality and filtered for length using mothur (v.1.23.1)^38^ trim.seqs command with the “oligos” parameter specifying a file that was used to remove the 16 S primer specific regions and demultiplex by the inline barcode. Additional quality trimming and filtering parameters were “flip = T, maxhomop = 10, bdiffs = 1, pdiffs = 4, qwindowaverage = 25, qaverage = 25, processors = 3, minlength = 400”. The sequence reads were then used as queries for a blastn search^39^ (parameters: -dust no -gapopen 0 -gapextend 0 -reward 1 -penalty -2 –word_size 10) of the CORE database^2^. They were assigned to species-level OTUs at the highest identity blast hit over 98% for an alignment length greater than 350 bp. Sequences without matches were clustered at 99.5% identity using uclust^40^, and non-chimeric clusters were identified using uchime^37^. Unmatched clusters were then curated with an approach that has been validated to accurately assign taxonomy to oral species or species groups in the CORE database^2^. Finally, OTU sequence frequencies were tabulated.

### Statistical analysis

The most common species were determined for inclusion in the groups shown in Fig. 1 using the following algorithm. Two steps of rarefaction were performed in order to account for biases of both library size and repeatedly sampled subjects. All samples were rarefied to the smallest library size, then rarefied samples from the same subject and age group were combined (counts were added) and rarefied again. This was repeated for a total of 1000 iterations. Species that were observed in at least 95% of these iterations were included and relative abundance boxplots for each species were plotted using the geom boxplot() function in the ggplot2 package in R.

NMDS of Bray-Curtis dissimilarities between samples was performed with the *metaMDS* function of the *vegan* package in R. Dispersion ellipses were calculated using the *ordiellipse* function of vegan, and PERMANOVA tests of differences between groups were calculated with the *adonis* function^41^. Shannon diversity was calculated using the *diversity* function of vegan. The average was taken from ten randomly rarefied samples of each sample (*rrarefy* in *vegan*), with the rarefaction level determined by the smallest sample. Differences between groups were determined with the Kruskal-Wallis one-way analysis of variance, and the effect of age was determined with a linear mixed effects model with the lmer function of the lme4 library in R^42^. Microbial community similarity among children over time was analyzed using the first dimension of a non-metric Multidimensional Scaling (MDS), and plotting the distance to the centroid against time. The relationship was analyzed using the linear mixed effects lmer function in R. The heatmap was generated in Excel based on pooling of all identified sequences from the indicated time intervals for the infants or all sequences for the adults. Presence/absence of the indicated species was based on random rarefication of the pooled sequences to the smallest pool size (498) to allow equal comparisons. Changes in bacterial species prevalence and community diversity over time were analyzed using a linear mixed effects model computed with the lmer function of the lme4 library in R^42^. Mann-Whitney Wilcoxon tests were performed with the wilcox.test function of R and p values were adjusted with the Benjamini-Hochberg false discovery rate correction using the p.adjust function to compensate for multiple comparisons. The decontam R package with DNA quantitation was used to screen for possible contaminant sequences^23^.

## Data Availability

The DNA sequences generated during and analysed during the current study are available in the NCBI SRA repository under BioProject PRJNA448135, SRA study SRP136797. https://www.ncbi.nlm.nih.gov/sra?linkname=bioproject_sra_all&from_uid=448135. Other data from the study is available from the corresponding author on reasonable request.

## References

[1] Dewhirst FE (2010). The human oral microbiome. J Bacteriol.

[2] Griffen AL (2011). CORE: a phylogenetically-curated 16S rDNA database of the core oral microbiome. PLoS One.

[3] Perez-Munoz ME, Arrieta MC, Ramer-Tait AE, Walter J (2017). A critical assessment of the “sterile womb” and “in utero colonization” hypotheses: implications for research on the pioneer infant microbiome. Microbiome.

[4] Dominguez-Bello MG (2010). Delivery mode shapes the acquisition and structure of the initial microbiota across multiple body habitats in newborns. Proc Natl Acad Sci USA.

[5] Cephas KD (2011). Comparative analysis of salivary bacterial microbiome diversity in edentulous infants and their mothers or primary care givers using pyrosequencing. PLoS One.

[6] Dzidic M (2018). Oral microbiome development during childhood: an ecological succession influenced by postnatal factors and associated with tooth decay. ISME J.

[7] Mason MR, Chambers S, Dabdoub SM, Thikkurissy S, Kumar PS (2018). Characterizing oral microbial communities across dentition states and colonization niches. Microbiome.

[8] Hohwy J, Reinholdt J, Kilian M (2001). Population dynamics of Streptococcus mitis in its natural habitat. Infect Immun.

[9] Kirchherr JL (2007). Physiological and serological variation in Streptococcus mitis biovar 1 from the human oral cavity during the first year of life. Arch Oral Biol.

[10] Caufield PW (2000). Natural history of Streptococcus sanguinis in the oral cavity of infants: evidence for a discrete window of infectivity. Infect Immun.

[11] Berkowitz, R. J. Mutans streptococci: acquisition and transmission. *Pediatr Dent* **28**, 106–109, discussion 192–108 (2006). 16708784

[12] Wan AK (2001). Association of Streptococcus mutans infection and oral developmental nodules in pre-dentate infants. J Dent Res.

[13] Kononen E (2000). Development of oral bacterial flora in young children. Ann Med.

[14] van Houte J (1994). Role of micro-organisms in caries etiology. J Dent Res.

[15] Socransky SS, Haffajee AD, Cugini MA, Smith C, Kent RL (1998). Microbial complexes in subgingival plaque. J Clin Periodontol.

[16] Gross EL (2012). Beyond Streptococcus mutans: dental caries onset linked to multiple species by 16S rRNA community analysis. PLoS One.

[17] Griffen AL (2012). Distinct and complex bacterial profiles in human periodontitis and health revealed by 16S pyrosequencing. ISME J.

[18] Aas JA (2008). Bacteria of dental caries in primary and permanent teeth in children and young adults. J Clin Microbiol.

[19] Koenig JE (2011). Succession of microbial consortia in the developing infant gut microbiome. Proc Natl Acad Sci USA.

[20] Palmer C, Bik EM, DiGiulio DB, Relman DA, Brown PO (2007). Development of the human infant intestinal microbiota. PLoS Biol.

[21] Lif Holgerson P, Ohman C, Ronnlund A, Johansson I (2015). Maturation of Oral Microbiota in Children with or without Dental Caries. PLoS One.

[22] Herrero ER (2016). Antimicrobial effects of commensal oral species are regulated by environmental factors. J Dent.

[23] Davis NM, Proctor DM, Holmes SP, Relman DA, Callahan BJ (2018). Simple statistical identification and removal of contaminant sequences in marker-gene and metagenomics data. Microbiome.

[24] Long SS, Swenson RM (1976). Determinants of the developing oral flora in normal newborns. Appl Environ Microbiol.

[25] Vorrasi J, Chaudhuri B, Haase EM, Scannapieco FA (2010). Identification and characterization of amylase-binding protein C from Streptococcus mitis NS51. Mol Oral Microbiol.

[26] Scannapieco FA, Solomon L, Wadenya RO (1994). Emergence in human dental plaque and host distribution of amylase-binding streptococci. J Dent Res.

[27] Nobbs AH, Jenkinson HF, Jakubovics NS (2011). Stick to your gums: mechanisms of oral microbial adherence. J Dent Res.

[28] Singh, A. K., Woodiga, S. A., Grau, M. A. & King, S. J. Streptococcus oralis Neuraminidase Modulates Adherence to Multiple Carbohydrates on Platelets. *Infect Immun* **85**, 10.1128/IAI.00774-16 (2017). 10.1128/IAI.00774-16 PMC5328485 27993975

[29] Cisar JO, Sandberg AL, Reddy GP, Abeygunawardana C, Bush CA (1997). Structural and antigenic types of cell wall polysaccharides from viridans group streptococci with receptors for oral actinomyces and streptococcal lectins. Infect Immun.

[30] Kolenbrander PE (1988). Intergeneric coaggregation among human oral bacteria and ecology of dental plaque. Annu Rev Microbiol.

[31] Palmer, R. J. Jr. *et al*. Interbacterial Adhesion Networks within Early Oral Biofilms of Single Human Hosts. *Appl Environ Microbiol* **83**, 10.1128/AEM.00407-17 (2017). 10.1128/AEM.00407-17 PMC5440702 28341674

[32] Yamane Kazuyoshi, Nambu Takayuki, Yamanaka Takeshi, Mashimo Chiho, Sugimori Chieko, Leung Kai-Poon, Fukushima Hisanori (2010). Complete Genome Sequence ofRothia mucilaginosaDY-18: A Clinical Isolate with Dense Meshwork-Like Structures from a Persistent Apical Periodontitis Lesion. Sequencing.

[33] Zhou Y (2016). Differential Utilization of Basic Proline-Rich Glycoproteins during Growth of Oral Bacteria in Saliva. Appl Environ Microbiol.

[34] Zhou, P., Li, X., Huang, I. H. & Qi, F. Veillonella Catalase Protects the Growth of Fusobacterium nucleatum in Microaerophilic and Streptococcus gordonii-Resident Environments. *Appl Environ Microbiol* **83**, 10.1128/AEM.01079-17 (2017). 10.1128/AEM.01079-17 PMC5601340 28778894

[35] Kononen E, Jousimies-Somer H, Asikainen S (1992). Relationship between oral gram-negative anaerobic bacteria in saliva of the mother and the colonization of her edentulous infant. Oral Microbiol Immunol.

[36] Kononen E, Kanervo A, Takala A, Asikainen S, Jousimies-Somer H (1999). Establishment of oral anaerobes during the first year of life. J Dent Res.

[37] Edgar RC, Haas BJ, Clemente JC, Quince C, Knight R (2011). UCHIME improves sensitivity and speed of chimera detection. Bioinformatics.

[38] Schloss PD (2009). Introducing mothur: open-source, platform-independent, community-supported software for describing and comparing microbial communities. Appl Environ Microbiol.

[39] Camacho C (2009). BLAST+: architecture and applications. BMC Bioinformatics.

[40] Edgar RC (2010). Search and clustering orders of magnitude faster than BLAST. Bioinformatics.

[41] Oksanen, J. B. F., Kindt, R. & Legendre, P. Package “vegan”. *Community ecology package*, *version* **2** (2013).

[42] Bates, D., Mächler, M., Bolker, B. & Walker, S. Fitting Linear Mixed-Effects Models Usinglme4. *Journal of Statistical Software* **67**, 10.18637/jss.v067.i01 (2015).

